# Developing a Healthy Environment Assessment Tool (HEAT) to Address Heat-Health Vulnerability in South African Towns in a Warming World

**DOI:** 10.3390/ijerph20042852

**Published:** 2023-02-06

**Authors:** Caradee Y. Wright, Angela Mathee, Cheryl Goldstone, Natasha Naidoo, Thandi Kapwata, Bianca Wernecke, Zamantimande Kunene, Danielle A. Millar

**Affiliations:** 1 Environment and Health Research Unit, South African Medical Research Council, Pretoria 0001, South Africa; 2 Department of Geography, Geoinformatics and Meteorology, University of Pretoria, Pretoria 0001, South Africa; 3 Environment and Health Research Unit, South African Medical Research Council, Johannesburg 2090, South Africa; 4 Department of Environmental Health, University of Johannesburg, Johannesburg 2000, South Africa; 5 School of Public Health, University of the Witwatersrand, Johannesburg 2000, South Africa; 6 The Public Health Agency, Johannesburg 2000, South Africa

**Keywords:** adaptation, climate change, environmental health, environmental indicators, global heating, heatwaves

## Abstract

Prolonged exposure to high temperatures can cause heat-related illnesses and accelerate death, especially in the elderly. We developed a locally-appropriate Healthy Environment Assessment Tool, or ‘HEAT’ tool, to assess heat-health risks among communities. HEAT was co-developed with stakeholders and practitioners/professionals from the Rustenburg Local Municipality (RLM), a setting in which heat was identified as a risk in an earlier study. Feedback was used to identify vulnerable groups and settings in RLM, consider opportunities and barriers for interventions, and conceptualize a heat-health vulnerability assessment tool for a heat-resilient town. Using information provided by the RLM Integrated Development Plan, the HEAT tool was applied in the form of eight indicators relating to heat-health vulnerability and resilience and areas were evaluated at the ward level. Indicators included population, poverty, education, access to medical facilities, sanitation and basic services, public transport, recreation/community centres, and green spaces. Out of 45 wards situated in the municipality, three were identified as critical risk (red), twenty-eight as medium-high risk (yellow), and six as low risk (green) in relation to heat-health vulnerability. Short-term actions to improve heat health resilience in the community were proposed and partnerships between local government and the community to build heat health resilience were identified.

## 1. Introduction

Heat waves are becoming a common phenomenon in South Africa [1,2] and are expected to become more frequent as global warming continues [3]. Temperature projections suggest that temperatures in South Africa are rising faster than in the rest of the world [4]. In South Africa, temperatures are projected to rise by 4 °C per year, making the occurrence of heat waves more likely [1]. Climate change is projected to increase both the duration (number of days) when health may be adversely affected by heat as well as the severity of the symptom levels and heat-related health risks in Africa [5].

Exposure to heat waves can cause significant morbidity and mortality [4]. Between 2018 and 2020, there were 3066 heat-related deaths worldwide [6]. In South Africa in 2016, from media reports, there were 11 heat-related deaths in the North-West Province [4], and 17 in the Northern Cape [7]. Heat-associated health effects include heat cramps, heat exhaustion, heatstroke, and hyperthermia [8]. Extreme high temperatures have also been associated with an increase in emergency hospital visits and admissions due to respiratory and cardiovascular diseases [9,10]. Temperature changes due to climate change affect the spread of diseases such as malaria [4]. Population groups most vulnerable to excessive heat exposure include the elderly, infants and children under 5 years old, people with pre-existing conditions, outdoor workers and people of low socioeconomic status [1,2]. 

Several South African studies have analysed the effects of heat on health considering different population groups, including various community settings and in the workplace [11,12,13,14,15,16]. Geographic areas of concern with the highest temperatures are dry regions in the north-western parts of the country with average temperatures of over 36 °C [3]. Heat health effects in major South African cities such as Durban, Cape Town, and Johannesburg demonstrate that early detection of temperature increases can mitigate the dangerous effects of rising temperatures [17]. Despite the published evidence, South Africa lacks surveillance systems to track and monitor the direct and indirect effects of excessive heat on human health and wellbeing [14]. 

South Africa has a wide range of communities, from suburban communities with formal housing to informal settlements constructed with substandard materials. Extreme heat stress and frequent storms increase the risk of death, homelessness, injuries, mental illness, diarrheal diseases, respiratory infections, and other negative health consequences, especially in informal settlements [18]. Equipping the more vulnerable communities with knowledge and resources to ensure successful adaptation to heat and extreme heat events is of utmost importance. In response to climate change and global warming, in 2011 South Africa launched its National Climate Change Response Plan White Paper (NCCRP) [12]. The NCCRP recognises heat stress as a key health and environmental risk [12]. The occurrence of excessive heat was highlighted in The South African National Climate Change and Health Adaptation Plan [18], with particular concerns over the risk of human injury and death due to informal housing and poor infrastructure quality. Thus, it has been recognised that people of low socioeconomic status are vulnerable to the detrimental impacts of excessive heat.

Death and illness due to heat and extreme heat events can be prevented [19]. The best prevention strategy necessitates an assessment of the risk and vulnerability of individuals and communities, as well as the planning of appropriate interventions [19]. The simplest practical prevention measures include, for example, shade, indoor cross ventilation, and the installation of mechanical cooling aids such as electric fans. Community awareness programmes are another means of preventing heat-associated health impacts. A health risk and vulnerability assessment tool can be used to help identify the greatest risks caused by climate change impacts to assist in the development and implementation of multiple preventative actions to protect human health and wellbeing [20]. To craft an assessment tool to analyse the vulnerability of a community to heat, an interdisciplinary approach is required which includes climatological information, the consideration of natural resources and the built environment, social factors, and communication between different stakeholders [21]. 

In 2013, a national climate change vulnerability assessment was conducted for South Africa that considered health risks that would be worsened by climate change, including communicable diseases, non-communicable diseases, vector-borne diseases, and heat related disease [22]. Several municipalities in South Africa have also conducted climate change vulnerability assessments for their municipalities, driving the development of climate change adaptation frameworks and strategies [23,24,25]. Though municipalities cover cities and suburbs, many informal communities are often not assessed and appropriate interventions for areas of even greater vulnerability cannot be planned.

To the best of our knowledge, no South African municipalities have adopted heat-health indicator tools to assess risks among communities living in their areas of authority. This study aimed to develop a locally-appropriate, simple heat-related health risk and vulnerability assessment for towns and municipalities to assess communities and areas in terms of heat-health risks. The risk tool findings would then be used to guide decision-making and planning, develop interventions, and protect communities from heat-health impacts. Annual increasing temperatures necessitate a vulnerability assessment tool that will assist low- and middle-income countries (LMICs) to plan and adapt for heat-health threats in their towns and municipalities.

## 2. Materials and Methods

### 2.1. Study Site

Rustenburg, one of the hottest towns in North-West Province [4], reported a 1% increase in average monthly minimum temperatures between 2001 and 2010 [26]. An earlier exercise (unpublished research) showed that Rustenburg was projected to be among the top ten towns where warming temperatures would occur in the coming decades. Hence, we decided to work in the town of Rustenburg in the Rustenburg Local Municipality (RLM).

**Figure 1:**
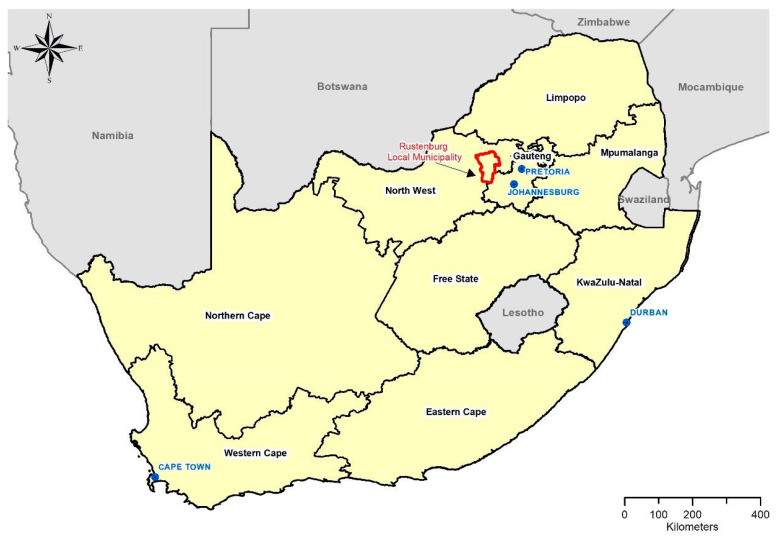
Location of Rustenburg Local Municipality in the North-West province of South Africa.

The RLM (Figure 1)—mainly an urban municipality—was established in 2000 [27] and is one of five sub-districts in the Bojanala District Municipality of the North-West Province. It is the most populous municipality in the province and the sixth most populous municipality in the country, with 29% of the total population within the Bojanala District Municipality [28]. 

### 2.2. Conceptual Heat-Health Risk Assessment Framework

In developing a heat-health risk assessment framework, we used the World Health Organization (WHO) guidelines for conducting health vulnerability and adaptation assessments [29], which were further adapted by the Ministry of Health in Canada [30]. The WHO guidelines for conducting a health and vulnerability assessment (not specifically for heat) entails five basic steps: (1) frame and scope the assessment; (2) conduct the vulnerability assessment; (3) future scenarios impact assessment; (4) adaptation assessment; and (5) monitor and manage risks.

The Ministry of Health Canada adapted the WHO guidelines and developed a framework for conducting vulnerability, impact and adaptation assessments at the community, regional or national levels for all climate change and health issues. Their framework proposed six steps to conduct a comprehensive assessment of vulnerability to heat (in the instance of our research) and places a strong emphasis on stakeholder engagement from the onset and throughout the assessment process. The six steps are to: (1) identify assessment scope, objectives and develop a work plan; and identify/organise stakeholders; (2) characterise heat exposure, community vulnerability, individual vulnerability; create an inventory of programmes and activities to address heat-health risks; evaluate the effectiveness of existing programmes and activities; (3) describe trends expected to influence heat-related health outcomes and projected increases in temperature and extreme events; (4) inventory possible adaptation options, prioritise adaptation options and assess barriers to adaptation options as well as how they can be overcome; (5) assess how changes in other sectors may influence heat-health risks; and (6) develop protocols for evaluating adaptation options, and develop protocols for monitoring the burden of heat-sensitive health outcomes.

**Figure 2:**
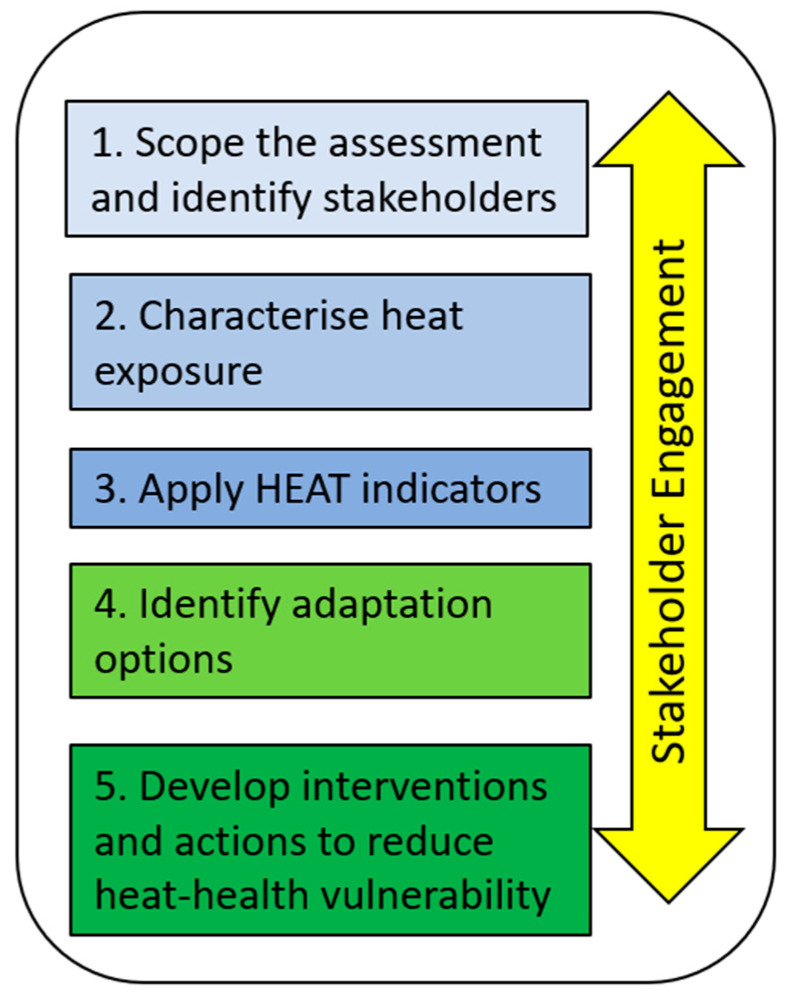
The steps we followed in an inclusive assessment of health vulnerability to heat events.

Using an adaptation of the WHO and Canadian guidelines, a framework was developed for conducting the heat-health vulnerability assessments at the municipal level for heat-related and health issues of interest. The framework entailed five steps (Figure 2) to conduct an inclusive assessment of health vulnerability to heat events and suggests a robust stakeholder engagement from inception throughout the assessment process.

### 2.3. The Integrated Development Plan

The vulnerability assessment should consider Statistics South Africa (where possible focussed on reports and not data to keep the task simple) and local government surveys for town profiling as well as literature reviews, grey literature reviews, epidemiological reviews, expert judgement, climate models, climate scenarios, environmental scans consisting of housing, schools and field visits. For this study, these data were predominantly available in a report entitled ‘The RLM Integrated Development Plan’ (IDP) [26]. The RLM IDP was read to understand comments made for the municipality and its wards and suburbs to identify indicators that we could apply in the Health Environmental Assessment Tool (HEAT). The facilities, activities, and services of the suburbs in each ward were drawn from the IDP by reading information about each ward and a detailed key illustrating the descriptors of each indicator was drawn up (Table 1).

### 2.4. Stakeholder Workshop

**Figure 3:**
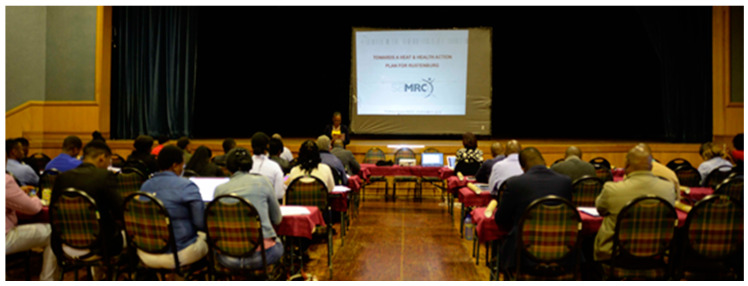
An RLM member of the mayoral committee opens the stakeholder workshop in Rustenburg with a brief address about the importance of this project (Permission obtained from participants to take the photograph at the workshop).

**Figure 4:**
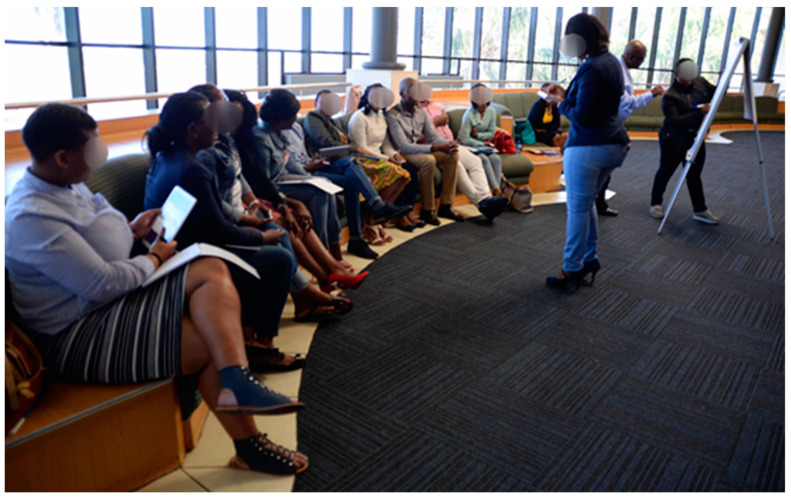
RLM members worked in a focus group to discuss and capture ideas about how extreme heat events affected the RLM community as well as provided their thoughts about heat-health indicators (Permission obtained from participants to take the photograph at the workshop).

The study’s main approach was stakeholder engagement with focus group discussions among RLM municipal and provincial officers. An interactive workshop was conducted at the RLM Civic Centre in October 2017 (Figure 3). The programme, structured into a morning plenary session followed by small group discussions and feedback (Figure 4), was designed to set the global context and then relate pertinent issues to the daily lives of people living and working in the RLM. The initial presentation highlighted the growing global concern around heat extremes and public health and offered international examples of heat protection interventions. This was followed by a presentation on key findings of a desktop review of heat and health in South Africa (available in a report from the corresponding author) as well as photographs of local people and places at risk from heat extremes in the RLM that were captured by the researchers in several pre-workshop site visits. 

During the workshop, information was sought from across sectors, including Environmental Health Practitioners and Health Promoters serving the RLM, as well as the other local municipalities in Bojanala District Municipality to provide a comprehensive picture of how climate change and extreme heat events affects the RLM community. 

Forty-eight participants engaged actively in five small group discussions to identify RLM-relevant circumstances and scenarios, including people, occupations, and places of increased heat vulnerability. Stakeholders recommended practical heat protective interventions. All material gathered from the stakeholder participants were their own opinions derived from their experience and knowledge. We also identified potential indicators from these discussions and considered these in light of the indicators we pinpointed in the RLM IDP for the HEAT tool.

A follow-up engagement workshop was held in February 2018 to give feedback, further the initial discussion and to engage senior managers and policy makers. Feedback was provided through an initial presentation followed by an open, lengthy discussion session. Stakeholders’ visions for a heat-resilient life in RLM were also gathered.

### 2.5. Tool Development and Application

Through the stakeholder engagement and with application of the conceptual framework described above, indicators for the HEAT tool were identified to assess heat and health vulnerability and resilience. “Traffic light” colours were used and consisted of critical risk (red), medium-high risk (yellow), and low risk (green). 

**Figure 5:**
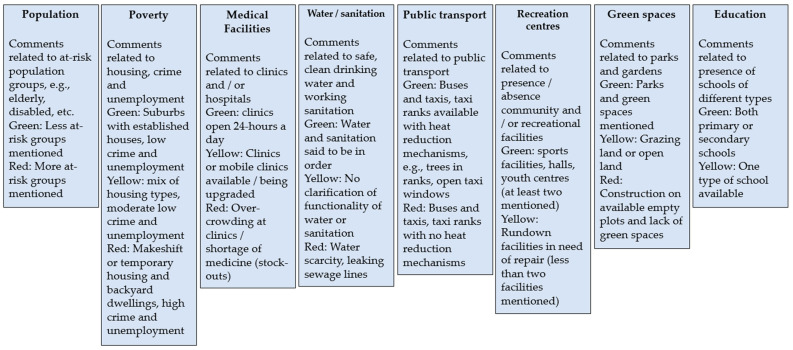
Description of HEAT heat-health indicators related to vulnerability and resilience. Some indicators do not have all three risk categories due to insufficient information to classify the comments and information in the IDP into more than two categories. In this instance, there will either be green and yellow (no red) or green and red (no yellow).

There were eight indicators identified from the IDP and stakeholder consultation to be a part of the HEAT tool (Figure 5). These indicators were developed by considering elements of vulnerability and resilience in relation to heat and health. The indicators were (1) population; (2) poverty; (3) education; (4) medical/health facilities; (5) water and sanitation services and provision; (6) public transport; (7) recreational/community centres; and 8) green spaces which promote cooling.

Using the RLM IDP and the literature, an initial assessment was made for each indicator for each ward. This was followed by expert consultation to consider the initial assessment of each suburb’s risk as a means of ground truthing the scoring exercise. Experts were drawn from the stakeholder workshop and contacted via email. Amendments were made when the expert provided additional information about the ward that required a change to the score. 

The risk for the ward was calculated as the average of the risks of each suburb in that ward. The greater the number of green blocks in each suburb, the lower the score for that ward. The lower the score for that ward, the lower the heat-health vulnerability.

For certain heat-related vulnerability indicators, it was not possible to draw out enough information from the IDP, the literature and expert consultation to delineate four risk categories (low—green, medium-high—yellow, and critical—red). These included the following indicators: population, education, public transport and recreational/community centres.

### 2.6. Calculation of Risk for Each Ward/Suburb

Based on the indicators and municipality characteristics, the heat-health vulnerability scores were calculated for each ward (Table 2 and Table 3). Risk scores were as characterised as low risk (green): 0.0 > *x* > 1.5; medium-high risk (yellow): 1.6 > *x* > 2.5 and critical risk: 2.6 > *x* > 3.0 (red).

As stated, data and information to assess the score for each indicator and for each ward was obtained from the RML IDP, the literature (where available) and expert consultation and scored as outlined in Figure 5. A detailed example of the scoring for an imaginary Ward ‘X’ is provided in Supplementary Table S1. The overall heat-health vulnerability for imaginary Ward ‘X’ is critical (red). This was calculated by scoring each indicator as red (3 points for high risk), yellow (2 points for medium risk) and green (1 point for low risk). The individual scores for each indicator were added up and then divided by the number of indicators (*n* = 8). The overall risk for Ward ‘X was 2.6 denoting a borderline critical risk. A detailed key is also provided to help illustrate the types of facilities, activities, and services in each category. In wards where insufficient information was provided to calculate the vulnerability, ‘NEI’ was used to denote ‘Not Enough Information’.

## 3. Results and Discussion

As empirical evidence on the degree and magnitude of global warming grows, failure to develop adaptation actions may leave vulnerable communities unprepared, consequently putting them at greater risk. It is imperative for decision-makers to envisage how global warming may increase the burden of diseases and to recognise vulnerable groups for feasible interventions to improve or create new policies and programmes to protect human health and wellbeing [8]. Thus, this study focused efforts on engaging stakeholders in assessing areas of heat-health vulnerability within their own communities and coming up with interventions and actions to reduce the impacts of heat, as well as increase resilience.

The RLM was chosen as an appropriate study area for developing the “HEAT” tool as the semi-arid area experiences high temperatures [26]. The high unemployment rate, high poverty levels and low levels of education exacerbate the risk of heat-related health impacts in the area [26]. RLM is divided into different wards that encompass different areas of authority [26]. Each ward is made up of one or more suburbs [26]. By highlighting the areas of greatest concern with respect to heat-health vulnerabilities allows for an effective and targeted implementation of interventions and preventative measures.

### 3.1. Heat-Health Risk Profile for Wards/Suburbs in the Rustenburg Local Municipality

The overall risk profile for the RLM indicated that most wards were at moderate-high risk (yellow) of adverse heat-related health impacts, with only a few suburbs at low risk (green) (Table 3). Thus, the risk spectrum shows very few suburbs in the RLM were protected from heat-health risks.

Three wards, namely wards 9, 10, and 40 (refer to Table 3) were categorised as being at critical risk. All three wards scored high in terms of unemployment rates and lacked recreational centres. Wards 9 and 10 had high risk populations such as children, people with disabilities and elderly people. Ward 40 had high risk housing and reported substance abuse problems. 

There were six high risk wards (comprising multiple suburbs each, some of which were informal settlements). On average, although there was reasonable access to water and sanitation, poverty and low-socioeconomic status made these wards more vulnerable. There were insufficient data gathered for Wards 6, 8, 23 and 42, which remained unclassified.

Studies on low-cost housing on South Africa’s East Coast show that indoor temperatures are typically 4 °C higher than outdoor temperatures [31]. There is usually a lack of appropriate roofing and insulation in indigent urban communities in South Africa, particularly in government-sponsored Reconstruction and Development (RDP) housing [32]. The potential health risk of high indoor temperature exposure, exacerbated by humidity, is likely to rise in the future [33]. Evidence of current and future negative health consequences will aid in the prioritization of coping mechanisms and the case for preventive policies [33].

### 3.2. Developing Interventions and Actions to Reduce Heat-Health Vulnerabilities

A settings approach was used to envisage heat-resilient spaces in which people can live, work, and play. Interventions were proposed by stakeholders from the RLM in five place-based categories: human settlements; taxi and bus ranks; marketplaces; schools; and parks, sport fields and stadia (Table 4). Several interventions were mentioned for each category, ranging from simple solutions such as trying to open windows and doors for cross-ventilation in a dwelling, to installation of a solar geyser which is deemed relatively costly. Mister sprays were suggested for use in public spaces such as minibus taxi ranks, however, given the concern around water scarcity in South Africa, the feasibility of such an intervention requires investigation. Water harvesting of rain and water storage in water storage containers called Jo-Jo tanks were considered as options for human settlements, schools and parks and sports fields. Awareness campaigns were also popular suggestions for all places to help raise awareness about the health risks of heat and actions that one can take to reduce these risks and subsequent impacts.

RLM stakeholders also proposed several short-term interventions to improve heat-health resilience in the RLM (Table 5). While some of these interventions are feasible for implementation in the short-term (e.g., hot weather awareness campaigns in parks and schools) others are medium- to long-term and require substantial resources (e.g., provide public swimming pools in schools and parks). Solutions also ranged from policy-related, such as changing the necessary distance between the construction of informal shacks, to personal behavioural actions, i.e., reducing time spent exercising outdoors when it is very hot.

Using the stakeholders’ vision for a heat-resilient RLM, a typical Heat Health Action Plan (HHAP) with a four-pronged approach was considered to activate steps and interventions to address heat-health vulnerability [34]. The HHAP includes “communications outreach” to educate people about the dangers of heat illness and how to avoid them [33]. Secondly, implementation of a “warning system” in the event of a heat wave, with actions mapped out for various role players [34]. Thirdly, training health care professionals to respond to heat illness. Finally, it is necessary to modify a town’s physical plan to better cope with heat, such as mapping high-risk areas, making potable water easily accessible, constructing temporary cooling spaces during heat waves, and long-term urban planning [34]. 

The HEAT tool aided in assessing the different geographic areas of the RLM and was used to help stakeholders decide what protective measures are required to mitigate heat-health risks. The sectors responsible for working together to build heat-health resilience in the RLM were also identified. Stakeholders also proposed that the public and private sectors must work together to ensure that the needs of vulnerable communities are met.

### 3.3. Study Limitations

We contacted as many role players and key stakeholders in the RLM and Bojanala District Municipality; however, it proved difficult to bring everyone together at the same time. Therefore, we had one large meeting and several email exchanges with those participants who were not able to attend the main workshop. There is the possibility of attendance bias for information provided by workshop attendees versus those participants who we spoke with via email. However, the majority of participants were at the workshop, so the effect is deemed minimal. Additionally, our approach with the workshop and focus group discussions was constrained by budget and the number of people from the RLM who attended the event. Future research should adopt a more thorough and comprehensive approach when conducting such qualitative research, as described by Linzalone et al. [35].

We drew our data to populate the indicators from the RLM IDP, which was published in 2017, the year of our study; however, it likely contained information about suburbs from one or two years prior, since the production of the IDP takes some months. There may have been subtle changes in some of the suburbs’ information although it is unlikely to have a marked difference on the outcomes of our assessment. Our data came from the comments and information presented in the IDP for each ward/suburb. All of this information was qualitative and descriptive; thus, it was not possible to have numerical or quantitative cut-offs or threshold values for the specific risk categories. We chose to use the IDP and not data (if it were available), since government practitioners and officials who will use the HEAT tool are unlikely to have access to data or have the resources to do the necessary calculations. We aimed to keep the tool as simple yet effective as possible. It was also difficult to reconcile differences in information about the same ward/suburb but for different HEAT indicators; again, it would be useful if actual data were available, but then, a researcher or consultant would be required to assist with the implementation of the assessment tool.

We chose to focus on adaptation and not mitigation in putting together and implementing the heat-health vulnerability assessment since mitigation tends to occur at international and national levels, and we were working at the town/local municipality level. Should the assessment tool be used for mitigation, it would require additional scoping.

## 4. Conclusions

The goal of this study was to demonstrate the applicability of collaboration with stakeholders when developing a Heat and Health Vulnerability Assessment tool for South African towns. Given the projected increases in ambient temperature, as well as the potential increase in heat waves for some areas of South Africa, it is critical that towns prepare for heat-related health impacts.

Inequality and poverty in South Africa have created a reduced capacity for heat adaptation among citizens in the lower socioeconomic income groups. Thus, efforts must be made for climate change preparedness during the early decision-making stages of planning and development. This study illustrates that there are areas of higher risk of heat vulnerability in RLM. Vulnerable groups, such as the elderly, infants and young children, as well as outdoor workers, are at risk of heat-related health effects. In addition, this study demonstrates the importance of creating a visual of heat-health risks so that the relevant authorities are better able to implement the tool and to advocate and facilitate change in vulnerable communities.

## Figures and Tables

**Table 1:** Description of HEAT indicators for those pertaining to heat-related vulnerability and resilience.

Indicator Category	Description
Vulnerability indicators	Population was assessed by mention of “elderly”, “disabled”, or “crèches” in the IDP.
	Poverty was estimated from the IDP by comments such as “high crime rate” and “high rates of unemployment” in each suburb. Areas with mention of makeshift housing and backyard dwellings were also considered low-income and classified as red; a mixture of dwelling types was yellow; and suburbs with established houses and suburbs were green.
Resilience/adaptive capacity indicators	‘Access to education’ was yellow or green for presence of schools in the suburb; green if there was mention of primary and secondary schools; and yellow if there was a mention of only one.
	‘Access to medical facilities’ was green if there were 24-h clinics; yellow if there were just the mention of clinics or mobile clinics or that they are being upgraded; red if there was overcrowding or a shortage of medicine identified for the suburb.
	‘Water and sanitation’ were yellow if mentioned but not clarified in terms of functionality; green if it was stated to be ‘safe, clean drinking water’ or working sanitation, and red if there are complications such as water scarcity, leaking sewage lines etc.
	‘Public transport’ was identified by considering if there was a mention of buses or a taxi rank, however classified as red if there was the presence of several of such public spaces which are high risk for heat-health impacts.
	‘Recreational/community centres’ included sports facilities, community halls, libraries, youth centres etc.; if more than two facilities existed, it was green. If they existed but were noted as being rundown or less than two existed, it was yellow.
	‘Green spaces’ applied where parks or green spaces were mentioned. Grazing land or open land was categorised as yellow. Some suburbs mentioned construction on available empty land, or a lack of green spaces and these suburbs were categorised as red.

**Table 2:** Description by heat-health vulnerability indicator and risk level in the HEAT tool.

Risk ^#^ Level	Population	Poverty	Education	Medical Facilities	Sanitation and Basic Services	Transport	Community Centres	Green Spaces
**High**	Elderly Disabled Crèches OR Unemployed	High crime rate Unemployment Need for RDP houses		Mobile clinic/clinic	Mention of any health threatening issue		Need for one: Youth centre Business centre/Community office park	No green space/empty available
		Informal or indigenous housing data	High drop-out rate	Insufficient supplies	(Sewage blockages or leaks)		Sports facilities/ground Library	
		Presence of migrants Classification of informal settlement		High rates of substance abuse			Community hall ORUpgrading any of the above	
**Medium**			One of the following:	Mobile clinic	Provision of basic services but functionality not specified		One of the following:	Grazing/open land
		RDP houses	Early learning centre	Ambulances	Mention of only one service (functional)	Scholar transport	Library Youth centre Business centre/Community office park	Parks to be upgraded or created
			Primary school	Upgrades to clinics	Service to be upgraded or installed		Sports facilities/Ground Community hall	
			High school FET * college				Upgrading of any of the above	
**Low**		Private land	Two or more of the following:		Provision of functioning sanitation and services:		One or more: Youth centre Business centre	
		Businesses	Early learning centre Primary school	Clinic OR 24-h clinic	Two or more services mentioned	Taxi rank	Community office park	Parks/green spaces
		Shopping centres	High school				Sports facilities/ground library	
			FET college				Community hall	

* Notes. FET: Further Education and Training. RDP houses: Reconstruction and Development Programme—low-cost house built and provided by the government. ^#^ Risk levels were denoted by colour as follows: High risk (red), Medium risk (yellow), Low risk (green)

**Table 3:** Calculated risk score for each ward in the RLM using the HEAT tool.

Ward No.	Suburb Names	Risk Score * (*x*)
1	Phatsima, Boshoek, Mefenya, Rasimone, Boekenhoutfontein, Magokgwane	1.7
2	Chaneng, Robega	1.9
3	Bafokeng North Mine, Impala, Luka Mogono, Rathibedi	1.6
4	Luka, Phokeng-Windsor	2.0
5	Sigmena, Lemenong Kwa Kgale, Lemenong, Lenatong, Punodung	1.8
6	Phokeng (Tshwara-Kotokoto), Saron, Dithabaneng, Masosobane, Masosobane 2, Salema, Phokeng, Ntsweng and Pitso, Greenside and Riverside, Makgokgwane, Ratshufi, Rafredi,	NEI
7	Babuanja, Lefaragatlha	1.7
8	Geelhoutpark Extensions 6.9 and 4, Mountain Ridge, Tlhabane West	NEI
9	Tlhabane	2.6
10	Tlhabane, Foxlake, Lebone, North-Flight	2.6
11	Jabula Hostel, Yizo, Oukasie	2.4
12	Meriting	1.4
13	Tlhabane, Oukasie-Sidzumo, Motsatsi, Lebone up to Dikgabong, Foxlake, Rustenburg North—Benoni, Berry	1.7
14, 15, 16, 17	Geelhoutpark, Protea Park, Boo Dorp, Cashan 1,2,3, Safari Garden 2,3,5,8, Rustenburg North-Benoni to Impala, Cashan Protea Park	1.5
18	Rustenburg East and North	1.8
19	Paardekraal, Sunrise Park	2.2
20	Boitekong Ext 4 and 2	1.6
21	Boitekong Ext	1.2
22	Kanana Hostel, Sunrise, Leshibidung, Mpho Khunou, Popo Molefe, Skeirlik, Mzanzi, Siza	2.0
23	Kanana, Mafike, Chachalaza	NEI
24	Freedom Park, Lemenong and Paardekraal Extension	1.5
25	Monnakato, Kopman, Rooikraal, Chaneng	1.9
26	Tananana, Tlaseng, Tsitsing, Maile Extension	2.3
27 and 28	Lethabong	1.7
29	Mabitse, Maumong, Barseba, Rankelenyane	2.1
30	Modikwe, Behtanie, Makolokwe	2.0
31	Marikana; Marikana Central Business district, Skierluk, Storm Huis, Swartkopies, Brampie Big House, Group Five, Burnely, Mahumapelo 1and2, Tlapa	1.5
32	Wagkraal, Suurplaat, Mmaditlhokwa, Marikana West, Retief, Mabomvaneng, Lapologang	1.7
33	Nkaneng; Bleskop Hostel; Ngawana Hotel	1.2
34	Mfidikoe, Zakhele, Entabeni Hostel, Bokamoso, Central Deep	2.2
35	Matebeleng, Ikemeleng, Thuane, Levus Bayer, Lekokjaneng, Bolane, Waterval	2.0
36	Cyferbuild, Boons, Breedsvlei, Naauwpoort, Modderfontein, Vlakdrift, Sandfontein, Dinie Estate, Sparkling Water, Molote, Mathopestad, Boshfontein	1.8
37	Jabula, Boitekong, Paardekraal, Sunrise Park, Sondela	2.4
38	Freedom Park, New Freedom Park	2.0
39	Ramotshanana	1.8
40	Boitekong, Chachalaza	2.7
41	Seraleng, Boitekong	1.7
42	Waterfall East	NEI
43	Jabula, Zinniaville, Karlienpark	1.8
44	Lekgalong, Ikageng, Serutube, Mafika, Mogajane, Lesung, Mosenthal, Marikana	1.9
45	Photsaneng, Thekwana, Nkaneng, Phula Mines, Karee Mines	2.0

Notes: * Risk scores as follows—low risk: 0.0 > *x* > 1.5; medium-high risk 1.6 > *x* > 2.5; and critical (high) risk: 2.6 > *x* > 3.0. In some wards, duplicate names of suburbs appear because different parts of the same suburb reside in a different ward. NEI = Not Enough Information.

**Table 4:** Interventions proposed by stakeholders in five categories for a heat-health resilient Rustenburg Local Municipality.

Human Settlements	Taxi and Bus Ranks	Marketplaces	Schools	Parks, Sports Fields and Stadia
Green building design	Existing taxi ranks	Remote areas	Cold, clean water to all	Athlete medical assessment
RDP housing	Increased shade	Use recyclable materials for structure and furniture	Green buildings, school competitions, school awards	Water fountains
Solar geysers	Cool coatings and non-heat absorbent material	Ventilation	Sunscreen for all learners and teachers	Provide sunscreen to athletes
Cross ventilation	Plant Trees	Subdivisions, e.g., food, arts and crafts	Harvest rainwater in Jo-Jo water storage tank	Installation of sprinklers at stadia and sports fields
Window size proportion to floor area	Water fountains in close proximity	Water-based recycling system	Heated/cooling floors and furniture	Careful location of stadia and sports fields
Orientation of windows	Mister sprays	Plant trees	Heat protection school uniforms	Water harvesting
High enough roofs (ventilation)	New taxi ranks	Public awareness campaigns	No hands water fountains	Adjusting to foreign environments
Use cool (also colour) roof materials	Dome type roofs	Licenses, permits	Indigenous and fruit/nut trees	No physical activity when hot and humid
Ceilings and insulation	Sufficient ventilation	Use gel-based stoves (clean cooking) data	No sweet drinks at school	
Light paint on walls			Awareness campaigns	
Trees for each dwelling			Food garden, some produce sold to community	
Communal swimming pool			Grey water harvesting	
			Give children trees to plant at home	

Note. RDP, Reconstruction, and Development Programme that began post-Apartheid to build low-costs houses for all people living in South Africa.

**Table 5:** Proposed short-term interventions for heat-health resilience in Rustenburg Local Municipality.

Intervention	Indicators	Possible Sector(s) Responsible
**Public Sector**		
Plant trees to replace those cut down to build houses	Home Affairs—mortality rate Health facility—mortality and morbidity related to heat Notifiable conditions related to heat, e.g., malaria Learners at schools Life expectancy Hospital records—people with heat stroke	Treasury Health Social development Planning Parks and recreation Housing Public Works
Provide water fountains in parks and taxi ranks		
Covered waiting areas at bus and taxi ranks		
Communal taps in streets to serve villagers		
Reduce queues and waiting times in clinics and SASSA pay points	Number of learners and elderly who faint—SASSA pay points	
Amendment and drafting of by-laws to address climate change	Funds allocated to preventative health	
Provide public swimming pools in parks and schools	Number of swimming pools—student performance	
Proper planning Location of schools Positioning of classrooms to minimize sun exposure	Learners at schools	
**Private Sector**		
Hot weather education and awareness campaigns	Lifestyle surveys (epidemiology to conduct)	NGOs and CBOs
Enforcement of cutting trees (stop), stopping veld fires and other activities contribute to air pollution/climate change	Advocacy campaigns—through IDP training and awareness programmes for environmental health	
Social mobility—people should be removed from high-risk areas to lower risk areas	Number of greening projects	
Insulation of corrugated iron houses or concrete with corrugated iron roofs by lining with cardboard	Improved and approved infrastructure—EHPs should approve	
Adequate space between shacks		
Staff rotation to reduce duration of exposure		Mining and industry
Introduction of flexible shifts to reduce exposure to heat (early morning—3:00 a.m. until 8 a.m. )		
Mines to rehabilitate mining areas and provide parks/greening, shade, swimming pools and other appropriate interventions as part of their corporate social responsibility programmes		
Compliance to building plans—natural, e.g., installation of airbricks to circulate air and artificial ventilation		
Remain indoors, plant trees outdoors for shade/Use sunscreen or umbrellas, wear hats		Individual
Wear breathable fabrics and light-coloured clothing because darker colours absorb heat		
Hydrate regularly by drinking enough water		

Note. CBOs, Community-Based organizations; EHPs, Environmental Health Practitioners; IDP, Integrated Development Planning; NGOs, Non-Governmental Organisations; SASSA, South African Social Security Agency.

## Data Availability

Data are available upon reasonable request of the corresponding author.

## Supplementary Materials

The following supporting information can be downloaded at: https://www.mdpi.com/article/10.3390/ijerph20042852/s1, Table S1: (a) Example assessments for Ward ‘X’ using HEAT and symbols that explain critical elements of each indicator, as identified in the Individual Development Plan. (b) A detailed key to help illustrate the types of facilities, activities and services in each category and used to calculate the risk score; Table S2: Description of HEAT indicators for those related to vulnerability and resilience; Figure S1. The six basic steps involved in an inclusive assessment of health vulnerability to life-threatening heat events.
