# Supplementary material for: Developing a Healthy Environment Assessment Tool (HEAT) to Address Heat-Health Vulnerability in South African Towns in a Warming World
Source: Int J Environ Res Public Health. 2023 Feb 6;20(4):2852. doi: 10.3390/ijerph20042852 (PMC9957206; doi:10.3390/ijerph20042852)
Supplement: Supplementary file 1 [file ijerph-20-02852-s001.zip › Supplementary Figure S1.pdf]

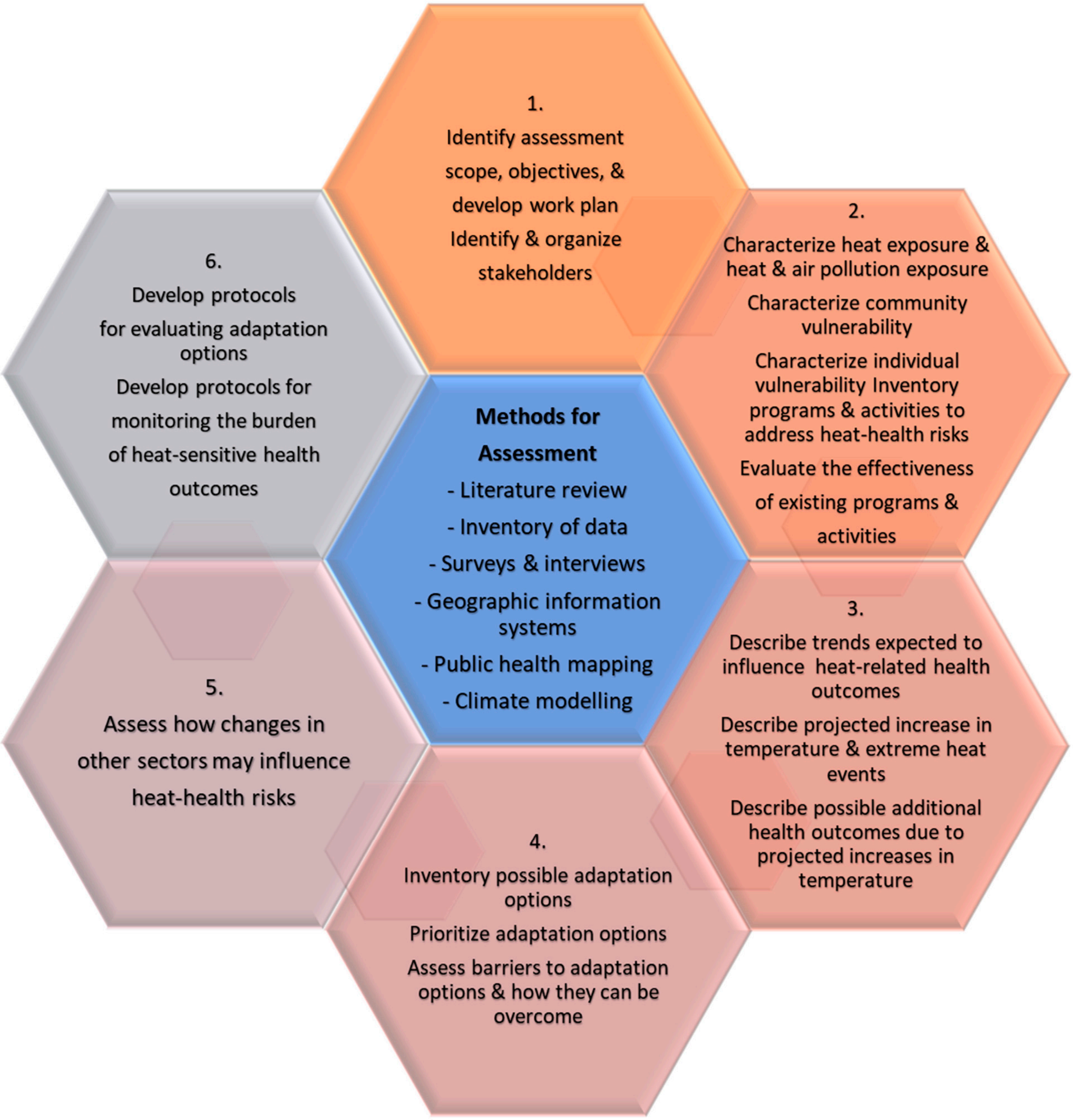

Figure S1. The six basic steps involved in an inclusive assessment of health vulnerability to life-threatening heat events.
