# Supplementary material for: Developing a Healthy Environment Assessment Tool (HEAT) to Address Heat-Health Vulnerability in South African Towns in a Warming World
Source: Int J Environ Res Public Health. 2023 Feb 6;20(4):2852. doi: 10.3390/ijerph20042852 (PMC9957206; doi:10.3390/ijerph20042852)
Supplement: Supplementary file 1 [file ijerph-20-02852-s001.zip › Supplementary Table S1.pdf]

Table S1. (**a**) Example assessment for Ward 'X' using the HEAT tool and symbols that explain critical elements of each indicator as identified in the RLM IDP. (**b**) A detailed key to illustrate the types of facilities, activities and services in each category used to calculate the risk score.

(a)

Risk	Heat-Health Vulnerability Indicators								Score
**Risk level assessed as:**	**Population**	**Poverty**	**Education**	**Access to medical facilities**	**Water and sanitation**	**Transport**	**Community Centres**	**Green spaces**	**Suburb/ward risk**
**High**									2.6
**Medium**									
**Low**									
**Score**	3	4	2	1	7	1	1	2	

(b)

Disabled	Business centres	Youth centre / Community centre / hall	Ambulance
Elderly	Marketplace / shops	Library	Unemployed or high drop-out rate
Children or orphans	Sports centre / recreational facilities	Toilets	Electricity
School	Mobile clinic	Sanitation and sewage	High crime / theft rate
RDP or informal houses	Business centres	Water	Green spaces, parks, or open land
Houses / private land	Clinic	Taxi rank or transport	Substance abuse
