# Supplementary material for: Developing a Healthy Environment Assessment Tool (HEAT) to Address Heat-Health Vulnerability in South African Towns in a Warming World
Source: Int J Environ Res Public Health. 2023 Feb 6;20(4):2852. doi: 10.3390/ijerph20042852 (PMC9957206; doi:10.3390/ijerph20042852)
Supplement: Supplementary file 1 [file ijerph-20-02852-s001.zip › Supplementary Table S2.pdf]

Table S2. Description of HEAT indicators for those related to vulnerability and resilience.

**Vulnerability indicators**	**Population** was assessed by estimated numbers of 'at-risk population groups' living in each suburb, including the elderly, disabled, or children, determined by the mention of "elderly", "disabled", or "crèches" in the IDP. It is not possible to give a threshold value, but proportions should be considered in relation to the adult group.
	**Poverty** was estimated from the IDP by comments such as "high crime rate" and "high rates of unemployment" in each suburb. Areas with mention of makeshift housing and backyard dwellings were also considered low-income and classified as red; a mixture of dwelling types was yellow; and suburbs with established houses and suburbs were green.
**Resilience / adaptive capacity indicators**	**'Access to education'** was yellow or green for presence of schools in the suburb; green if there was mention of primary and secondary schools; and yellow if there was a mention of only one.
	**'Access to medical facilities'** was green if there were 24-hour clinics; yellow if there were just the mention of clinics or mobile clinics or that they are being upgraded; red if there was overcrowding or a shortage of medicine identified for the suburb.
	**'Water and sanitation'** were yellow if mentioned but not clarified in terms of functionality; green if it was stated to be "safe, clean drinking water" or working sanitation, and red if there are complications such as water scarcity, leaking sewage lines etc.
	**'Public transport'** was identified by considering if here was a mention of buses or a taxi rank, however, classified as red if there was the presence of several of such public spaces which are high risk for heat health impacts.
	**'Recreational / community centres'** included sports facilities, community halls, libraries, youth centres etc. If more than two facilities existed, it was green. If they existed but were rundown or less than two existed, it was yellow.
	**'Green spaces'** applied where parks or green spaces were mentioned. Grazing land or open land was classified yellow. Some suburbs mentioned construction on available empty land, or a lack of green spaces and these suburbs were classified red.
